# The Swedish translation of Perceptions of Restraint Use Questionnaire (PRUQ): A test-retest reliability study in two dementia nursing homes

**DOI:** 10.1186/s12877-021-02486-2

**Published:** 2021-10-22

**Authors:** Charlotta Thunborg, Martin Salzman-Erikson, Annakarin Olsson

**Affiliations:** 1 Karolinska Institutet Department of Neurobiology, Care Sciences and Society, Clinical Geriatrics, Stockholm, Sweden; 2 School of Health, Care and Social Welfare, Division of Physiotherapy, Mälardalen University, Västerås, Sweden; 3 Theme Inflammation and Aging, Karolinska University Hospital, Stockholm, Sweden; 4 Faculty of Health and Occupational Studies, Department of Caring Sciences, University of Gävle, Gävle, Sweden

**Keywords:** Restraint, Geriatric Care, Attitude, Reliability

## Abstract

**Background:**

The Perceptions of Restraint Use Questionnaire measures perception of restraint in a 17-item questionnaire. The aim of this study was to assess the test-retest reliability of the PRUQ as a measure of staff attitudes to restraint in elderly older persons care for people with dementia from two different nursing homes, and its ability to produce reliable results.

**Methods:**

Twenty-six staff members from two different nursing homes completed the 17-item PRUQ twice with 14–21 days between time points. As the questionnaire has already been translated in another study, the current study evaluated total item scores, mean, internal consistency, and intraclass correlation for reliability purposes.

**Results:**

The internal consistency Cronbach’s Alpha were ˃ ≥0.726. The Intraclass correlation (ICC) between test and retest was moderate to good for the three subscales, with ICC (A,1) and ICC (C,1) values approximately equal and in the range 0.480–0.962. A Bland-Altman plot of the PRUQ total mean scores illustrates no systematic change in the mean.

**Conclusions:**

The Swedish version of the PRUQ shows mainly good reliability. Therefore, we suggest that researchers continue to develop the PRUQ to be an even higher reliable questionnaire of health care professionals’ perceptions of measure for restraint use in nursing homes for persons with dementia.

## Introduction

Older people living in nursing homes often have cognitive deficits. This can in its turn increase the risk of falls, self-harm and agitation [1]. To meet the challenges that cognitive deficits may pose, staff intervene in various ways to prevent such behavior. For example, items that may be used to do harm could be removed. However, verbal interventions are also used when encountering people with cognitive deficits [2, 3]. In some instances, verbal interventions are enough and physical restraint is described as ‘a last resort’ in the literature [4, 5]. Applying mechanical/physical restraints restraint has been reported be to ‘part of the job’ but are also perceived as highly controversial [6, 7]. Physical restraints are frequently used in the care of elderly people older persons in many countries; studies in nursing homes have reported that prevalence levels range from 41 to 64 % [8]. Physical restraint is defined as any manual restraint method, including physical or mechanical devices, material or equipment immobilising or decreasing the ability of a patient to move arms, legs, body, or head freely in all hospital settings [9]. The use of restraints is associated with identifiable negative outcomes such as worse physiological and psychological condition [10, 11]. Physical restraint is a procedure commonly used in institutional long-term care of old people. However, it has been questioned both from an ethical perspective, and because there is a lack of sound evidence of their effectiveness [10]. Restraint use often includes full side rails, vests, waist belts and ankle restraints [12]. Restraints can be perceived as coercive and have been subjected to intense debate during recent decades. In some countries, regulations have been developed in order to reduce their use. In 2017 in Sweden, The National Board of Health and Welfare drew up national guidelines as well as a series of measures that decreased the overall rate of restraint usage in dementia long-term care settings [13]. In other countries, however, progress in this area has been slower. Studies in Western countries have estimated that 13–20 % of elderly patients older persons admitted to hospitals experience some form of physical restraint during their stay. One study showed that prevalence ranged from 6 to 31 % and that the prevalence of residents with at least one physical restraint was 26.8 % [14]. Furthermore, a systematic review showed that physical restraint is associated with mortality in nursing home residents [15]. Due to the limited research in this area, it is needed to further understand physical restraint procedures. In order to provide an understanding of how health care professionals apply restraints, reliable questionnaires are required.

## Aim

The aim of this study was to test the reliability and internal consistency of the Swedish version of Perceptions of Restraint Use Questionnaire (PRUQ), among staff working in nursing homes for people with dementia.

## Methods

### Procedures

Before the study was conducted, the first author contacted the original authors of The Perceptions of Restraint Use Questionnaire (PRUQ), Dr Lois Evans & Dr Neville Strumpf, United States, and they gave their approval for a reliability study of the Swedish translation. The PRUQ, (Table 1) was developed by Evans and Strumpf (1993) [11] to determine the relative importance caregivers ascribe to reasons for using physical restraints with the elderly older persons. Questionnaire translation was conducted in another research project [16], and content validity, internal consistency, and total item score correlation have been presented in several previous studies [17–20]. Based on a convenience sample, two nursing home managers were contacted and informed about the aim of the study. The clinical convenience sample, which is a type of nonprobability sampling, was based on earlier research collaborations with the first author. After receiving information, the nursing home managers gave their permission to test the reliability of the PRUQ at their units.

**Table 1:** Perceptions of Restraint Use Questionnaire (PRUQ)

Q1 Protecting an older person from falling out of bed?
Q2 Protecting an older person from falling out of chair?
Q3 Protecting an older person from unsafe ambulation?
Q4 Preventing an older person from wandering?
Q5 Preventing an older person from taking things from others?
Q6 Preventing an older person from getting into dangerous places or supplies?
Q7 Keeping a confused older person from bothering others?
Q8 Preventing an older person from pulling out catheter?
Q9 Preventing an older person from pulling out a feeding tube?
Q10 Preventing an older person from pulling out an IV line?
Q11 Preventing an older person from breaking open sutures?
Q12 Preventing an older person from removing a dressing?
Q13 Providing quiet time or rest for an overactive older person?
Q14 Providing for safety when judgment is impaired?
Q15 Substituting for staff observation?
Q16 Protecting staff or other patients from physical abusiveness/combativeness?
Q17 Managing agitation?

### Data collection

Data collection took place from April 2017 to September 2017. Two co-investigators working at the nursing homes distributed the envelopes with the questionnaires twice, with 14 to 21 days between time points, to the health professionals in the nursing homes. The co-investigators worked as an occupational therapist and as the head nurse in charge of care at the nursing homes. They received information about the aim, the PRUQ and the theories of reliability, to be able to answer questions from participants. Written instructions to staff completing the PRUQ was formulated according to the appendix K [21]. *In caring for the older adult, physical restraints are sometimes used. Such restraints include vests, belts or sheet ties, crotch or diaper restraints, ankle or wrists ties, hand mitts, or locked geriatric chairs with fixed tray tables. Following are reasons sometimes given for restraining older people. In general, how important do you believe the use of physical restraints are for each reason listed? (Please circle the number that represents your choice 1 = not important to, 5 = very important).* The envelopes also contained a letter with information about the purpose of the study and information regarding confidentiality. After they were completed, the co-investigators sealed the envelopes and redistributed the completed questionnaires back to the first author.

### The Perceptions of Restraint Use Questionnaire

PRUQ was developed by Evans and Strumpf in 1988 [22] and revised in 1993 [11]. It lists 17 of the most often cited reasons for using restraints. The items are assessed using a Likert scale, ranging from 1 (not important) to 5 (very important). A higher score indicates that the person is more prone to using restraints. The items are summed to an index and divided by 17, giving a range from 1 to 5. The internal consistency value (Cronbach alpha) of the scale has been reported to be 0.94 to 0.96 [11]. Face validity and content validity were evaluated by a panel of five gerontologic nurse experts [22]. The coefficient alpha was 0.80 with 18 professional hospital nurses and 0.74 with a sample of 20 nursing home staff [23]. During the period 1987–1993, the instrument was updated to include more items regarding fall risk and treatment interference. In a 1993 study including 184 European nursing personnel, the latest version had a coefficient alpha of 0.96 [11]. In related literature, no cut-off value has been indicated. The mean score is calculated for the whole questionnaire by summing the value of each answer (ranging from 17 to 85 points) and dividing by 17. Higher values indicate that the situation is considered an important justification for using physical restraints, and vice versa. The PRUQ was initially developed for an acute care hospital setting and is not readily adapted for use with people with dementia living in nursing homes. The factor structure used in this study is based on factor loadings from a Spanish study in eight emergency hospitals and 19 nursing home facilities [17]. They are categorized into three factors (i) providing safe environment (F1), (ii) prevention of therapy disruption (F2) and, (iii) prevention of falls (F3). (i) prevention of falls (F3), (ii) prevention of therapy disruption (F1) and (iii) providing a safe environment (F2), see Table 2. We wanted to find out whether the factor structure of the Swedish translated tool was the same as the factor structure proposed for the Spanish versions. This might be helpful in future studies, if there is a need to perform measurement invariance analyses and evaluate if the constructs of the PRUQ are measured equally well in different groups or if their measurement differs substantially.

**Table 2:** Three factors with the corresponding items of the PRUQ

**F1: Providing safe environment:**
Q4. Wandering
Q5. Taking things from others
Q6. Getting into dangerous places or supplies
Q7. Bothering others if confused
Q13. Quiet time or rest if overactive
Q14. Safety when judgement impaired
Q15. Substituting staff observation
Q16. Physical abusiveness/combativeness
Q17. Managing agitation
**F2: Prevention of therapy disruption:**
Q8. Pulling out a catheter
Q9. Pulling out a feeding tube
Q10. Pulling out an intravenous line
Q11. Breaking open sutures
Q12. Removing a dressing
**F3: Prevention of falls:**
Q1. Falling out of bed
Q2. Falling out of chair
Q3. Unsafe ambulation

### Setting and subjects

In this study both nursing staff and paramedical health care professionals participated (e.g., occupational therapists, physiotherapists). A sample of 32 staffs (*n* = 10 and *n* = 22 from the two settings; three men), accepted to participate in the study. The staff’s mean age was 39.0 (± 12.0) years, and they had an average of 10.5 (± 7.9) years of experience in care of older persons. Test-retest reliability was examined for 26 caregivers out of 32 included on day 1, and then 14–21 days after the first rating.

### Statistical analysis

All statistical analysis was performed with IBM SPSS 27.0 (SPSS Inc., Chicago, USA). Descriptive data are presented as mean (SD), min-max, number (n). Cronbach’s alpha coefficient was used for internal consistency. This is considered adequate when values greater than 0.7 are obtained [24]. To establish the relative reliability, the intraclass correlation coefficients ICC(A,1) (absolute agreement) and ICC(C,1) (consistency) were calculated [25]. The two coefficients provide complementary information about the reliability of the method. Both should be reported together with their confidence intervals. The estimate of the reliability where the effect of bias is taken into account is provided by ICC(A,1), while ICC(C,1) neglects this effect [26]. The individual mean substitution method was used at the scale level to compensate for missing values [27].

### Reliability

Reliability is defined as the extent to which measurements can be replicated. Intraclass correlation coefficient (ICC) is a widely used reliability index in test-retest, intra-rater, and interrater reliability analyses. According to Shrout and Fleiss (1979) [24] some important topics should be considered when using the ICC approach. Firstly, the design should not be a correlational study, but a reliability study with the objective to detect the effect of various sources of errors. Factors that may generate measurement errors, such as time or rater, should be taken into consideration. The test-retest reliability was measured with intraclass correlation coefficient (ICC) analyses [25], which were performed separately for the three domains and for each of the total of 17 items owing to the structure of the PRUQ. Ninety-five per cent confidence limits were calculated for the ICCs. Single factor ANOVA was performed for calculating F-value and p-value, where the mean scores of PRQ are considered the dependent variable, with levels ranging from 1 to 5, and the between-days are considered the independent variable, ranging from 14 to 21 days.

### Ethical considerations

Detailed information, including a description of the PRUQ scale and of the pseudonymization data handling, was provided in an information sheet when the staff members were asked to participate. The subjects who returned the completed questionnaire were considered to have consented. This research is not considered as human subject research according to Swedish law (SFS) and thus, no ethical approval was required. This was because the research did not concern any personal questions regarding the nurses’ health or addressed any other sensitive topics, nor was any personal information kept on file. The two co-investigators informed the health care professionals during a weekly team meeting and the envelopes were handed out to those interested. This was an anonymous survey and the subjects’ privacy was protected. The aim of the study and the way that the results would be used were described to the staff at the beginning of the questionnaire. An explanation was given indicating that the results would be used only for statistical processing.

## Results

**Fig. 1:**
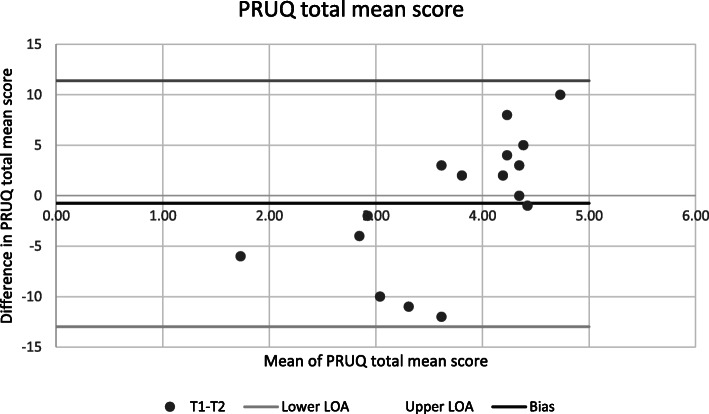
Bland-Altman Graph presenting The Perception of Restraint use Questionnaire total mean score

Data from 26 staff out of 32 were incorporated after excluding those that did not meet inclusion criteria (*n* = 6 with incomplete data on more than 50 % of the items of the PRUQ). The item mean substitution method was used at the scale level to compensate for missing values. The mean score on the PRUQ was 3.69 (possible range 1–5, item mean (SD) values ranged from 1.73 to 4.73 (0.80–1.64), see Tables 3 and 4. The results of the reliability analysis are shown in Tables 3, 4 and 5. Internal consistency was sufficient with Cronbach’s Alpha 0.918 0.926 for the total score, see Table 5. However, the three factors (subscales) had one subscale (F3) with Cronbach alpha clearly below 0.90, see Table 5. The relative test-retest reliability of the different PRUQ showed ICC (A,1) (absolute agreement) values ˃ ≥0.726. The ICC (C,1) (consistency) values were close to the corresponding ICC (A,1) values, see Table 5. A Bland Altman plot of the PRUQ total mean score illustrates that there was no systematic change in the mean (Fig. 1).

**Table 3:** Mean, standard deviation (SD), and difference in mean and Intraclass correlation

PRUQ (Item)	Mean T1	SD	Mean T2	SD	Diff. in mean T1-T2	ICC (CI 95 %)
**1**	4.35	1.16	4.35	1.02	0.15	0.665 (0.253–0.850)
**2**	4.42	1.14	4.46	0.99	0.15	0.689 (0.306–0.860)
**3**	4.23	1.48	3.92	1.35	0.12	0.838 (0.638–0.927)
**4**	1.73	0.92	1.96	0.99	-0.08	0.712 (0.357–0.871)
**5**	2.85	1.41	3.00	1.33	0.08	0.828 (0.617–0.923)
**6**	4.73	0.83	4.35	1.23	-0.40	0.776 (0.500-0.899)
**7**	2.85	1.22	3.00	1.23	-0.01	0.798 (0.548–0.909)
**8**	4.23	1.14	3.92	1.44	-0.30	0.822 (0.603–0.920)
**9**	4.38	1.06	4.19	1.13	-0.07	0.909 (0.797–0.959)
**10**	4.19	1.27	4.12	1.34	-0.07	0.897 (0.770–0.954)
**11**	4.35	1.13	4.23	1.31	-0.18	0.821 (0.600–0.920)
**12**	3.62	1.17	3.50	1.45	-0.28	0.834 (0.630–0.926)
**13**	3.31	1.38	3.62	1.63	-0.25	0.790 (0.531–0.906)
**14**	3.62	1.44	4.08	1.26	0.18	0.557 (0.013–0.802)
**15**	3.04	1.71	3.42	1.60	0.10	0.829 (0.618-0.0923)
**16**	3.81	1.60	3.73	1.43	0.17	0.862 (0.693–0.938)
**17**	2.81	1.63	3.00	1.67	-0.05	0.920 (0.824–0.964)

**Table 4:** Descriptive for PRUQ direct scores between test 1 and test 2 (F1, F2 and F3)

*N* = 26	TEST 1			TEST 2		
	**Mean**	**SD**	**Min-Max**	**Mean**	**SD**	**Min-Max**
Total	3.69	1.47	1–5	3.71	1.44	1–5
F1*: (Q4, Q5, Q6, Q7, Q13, Q14, Q15, Q16, Q17)	3.21	1.55	1–5	3.36	1.52	1–5
F2*: (Q8, Q9, Q10, Q11, Q12)	4.14	1.17	1–5	4.24	1.13	1–5
F3*: (Q1, Q2, Q3)	4.35	1.16	1–5	4.02	1.30	2–5

*F1 Providing safe environment, *F2 Prevention of therapy disruption, *F3 Prevention of falls

**Table 5:** Cronbach’s alpha and intraclass correlation (ICC(A.1) and ICC(C.1)) (95 % CI) for the PRUQ, three factors

	Cronbach’s Alpha	ICC (A,1)	95 % CI	ICC (C,1)	95 % CI	ƒ value	*p* value
Total	0.926	0.862	0.717–0.936	0.926	0.835–0.967	0.005	0.942
F1* (Q4, Q5, Q6, Q7, Q13, Q14, Q15, Q16, Q17)	0.914	0.842	0.679–0.926	0.914	0.809–0.962	1.65	0.211
F2* (Q8, Q9, Q10, Q11, Q12)	0.912	0.837	0.674–0.923	0.912	0.805–0.960	1.12	0.300
F3* (Q1, Q2, Q3)	0.851	0.726	0.480–0.867	0.842	0.648–0.929	2.96	0.097

*F1 Providing safe environment, *F2 Prevention of therapy disruption, *F3 Prevention of falls

## Discussion

In the present study, internal consistency and ICC analysis of the measure of PRUQ were examined in a sample of 26 health care professionals working in two nursing homes. We presented the results from our analysis on item level as well as in a factor structure originally based on factor loadings analysis from a Spanish study carried out in eight emergency hospitals [17]. ICC is usually found to have a value between 0 and 1 and refers to correlations within a class of data, for example an assessment scale with different items related to a concept of interest, e.g., attitudes [28]. Our psychometric analyses of PRUQ from 26 health care professionals’ ratings produced high internal consistency analysed with Cronbach’s alpha ˃ 0.85, and moderate to good ICC was observed for F2-*preventing therapy disruption* and F3-*preventing falls*, and for F1-*providing a safe environment* where the ICC value was 0.842 which is considered good reliability. The alpha coefficient is calculated by averaging the coefficients that result from all variances to the general variances and which ranges from 0 to 1. Further the alpha coefficient examines whether the items in an assessment scale are related to a same concept of interest (i.e., have the integrity to explain a homogenous structure). i.e., are the different items a A Cronbach’s alpha coefficient between 0.60 and 0.80 indicates a good reliable scale and a Cronbach’s alpha coefficient between 0.80 and 1.00 is considered excellent reliable [29]. ICC values above 0.8 or 0.9 are often regarded as a sign of good or excellent reliability [24] which was the case for F1*providing safe environment* and F2-*preventing therapy disruption*. However, whether an ICC value is acceptable depends on the intended use of the instrument [26].

Another important factor for reliability is the missing data substitution method. In this study, after removing cases with more than 50 % missing data, the final data set (n = 26) only had four missing data points. We therefore considered the individual’s mean as an appropriate and simple method for dealing with missing data [27]. Since scale reliability depends on correlations among the values in the scale, the wrong mean substitution method severely could underestimate the reliability if the study has more than a few cases with missing data [30].

As an extra safeguard, we also analysed the between-days F-value. which was F = 1,12 with *p* = 0,30, F = 2,96 with *p* = 0,097 and F = 1,65 with *p* = 0,21 for F2-*preventing therapy disruption* and F3-*preventing fall* and F1-*providing a safe environment*, respectively. This low F-value and relatively high p-value show that the systematic differences between days are not significant. The reported low F-values and relatively high p-values show that the systematic differences between days are not significant. The Spanish study [17] reported very similar internal consistency reliability coefficients (Cronbach’s alphas) for the three factors to the ones reported by our study, even though the design of our study was different. The Spanish study was designed to explore the gaps that exist in the PRUQ internal structure and measurement invariance across different groups of respondents. In our study the internal consistency of the 3 factors showed 0.914 (F1), 0.912 (F2) and 0.851 (F3), respectively, while the Spanish study showed 0.94, (F1), 0.95 (F2) and 0.85 (F3) [17]. In the Japanese study the internal consistencies of PRUQ was 0.91 for nurses, 0.92 for care workers [18], and in a study from Australia the test–retest reliability of the PRUQ was ICC 0.88 [19].

Further, analysis of the first and second administration of the PRUQ found that the Cronbach’s alpha coefficient was 0.926, indicating a strong internal consistency between the total scores from the two administrations.

## Limitations

Conducting the study in two small nursing home wards was a limitation. Health care professionals at these two nursing home settings may not represent all health care professionals in Sweden. Additionally, participation of staff was limited to those interested, which in turn may have led to a selection bias as it is possible that the participating staff members’ attitudes to restraint may differ from those of staff not interested in the research. Another issue of concern relates to a problem identified with the PRUQ. Although this instrument has been well-validated in the literature, results from the ICC analysis in F3- *prevention of falls* showed moderate results, indicating that the instrument might not have produced valid or reliable results in this setting and may also reflect the problems with staff knowledge that affect attitudes and perceptions. On the other hand, on an item level, item-14 *safety when judgement impaired* was the only item resulting in poor ICC = 0.557 with 95 % CI (0.013–0.802).

## Conclusions

The PRUQ was initially developed for an acute care hospital setting and is not readily adapted for use with people with dementia living in nursing homes. However, the Swedish version of PRUQ shows preliminary evidence for reliability and can be used to assess health care professional’s perception of restraint use in nursing homes for persons with dementia. The PRUQ has potential to be used as a tool to assess attitudes and perceptions to restraint use and reflect the complexity of quality of care and protection for persons with dementia.

## Data Availability

The datasets generated and/or analysed during the current study are available from the first author on reasonable request.
